# Survival of Dopaminergic Amacrine Cells after Near-Infrared Light Treatment in MPTP-Treated Mice

**DOI:** 10.5402/2012/850150

**Published:** 2012-05-30

**Authors:** Cassandra Peoples, Victoria E. Shaw, Jonathan Stone, Glen Jeffery, Gary E. Baker, John Mitrofanis

**Affiliations:** ^1^Discipline of Anatomy & Histology F13, The University of Sydney, Sydney, NSW 2006, Australia; ^2^Discipline of Physiology F13, The University of Sydney, Sydney, NSW 2006, Australia; ^3^Institute of Ophthalmology, University College London, London EC1VGEL, UK; ^4^Department of Optometry and Visual Science, City University, London EC1V7DD, UK

## Abstract

We examined whether near-infrared light (NIr) treatment (photobiomodulation) saves dopaminergic amacrine cells of the retina in an acute and a chronic 1-methyl-4-phenyl-1,2,3,6-tetrahydropyridine (MPTP) mouse model of Parkinson disease. For the acute model, BALB/c mice had MPTP (100 mg/kg) or saline injections over 30 hours, followed by a six-day-survival period. For the chronic model, mice had MPTP (200 mg/kg) or saline injections over five weeks, followed by a three-week-survival period. NIr treatment was applied either at the same time (simultaneous series) or well after (posttreatment series) the MPTP insult. There were four groups within each series: Saline, Saline-NIr, MPTP, and MPTP-NIr. Retinae were processed for tyrosine hydroxylase (TH) immunochemistry, and cell number was analysed. In the MPTP groups, there was a significant reduction in TH^+^ cell number compared to the saline controls; this reduction was greater in the acute (*~*50%) compared to the chronic (*~*30%) cases. In the MPTP-NIr groups, there were significantly more TH^+^ cells than in the MPTP groups of both series (*~*30%). In summary, we showed that NIr treatment was able to both protect (simultaneous series) and rescue (posttreatment series) TH^+^ cells of the retina from parkinsonian insult.

## 1. Introduction

Many previous studies have reported that mitochondrial dysfunction is a key component of the pathogenesis of Parkinson disease, a striking motor disorder that develops after a major loss of dopaminergic cells in the substantia nigra pars compacta (SNc) [1]. Hence, treatments that target the protection and/or enhancement of mitochondrial function against insult may prove to be useful therapeutic tools. One such treatment is low intensity light therapy, known also as photobiomodulation or near infrared light (NIr) treatment. Previous studies have shown that when exposing cells to NIr treatment, mitochondrial function and ATP (adenosine-5′-triphosphate) synthesis are enhanced considerably [2, 3]. Although the precise mechanism is not clear, it has been reported that NIr treatment benefits overall cell function (and limits toxic insult) by not only decreasing reactive oxygen and nitrogen species, but also increasing ATP content and production of specific cytokines in cells. NIr treatment is thought to increase electron transfer in the respiratory chain and activation of photoacceptors, such as cytochrome oxidase, within the mitochondria [2, 3].

In this study, we sought to extend our previous findings on the SNc of MPTP-treated mice [4, 5] by exploring whether NIr treatment enhances the survival of tyrosine hydroxylase (TH)^+^ dopaminergic cells located in the retina. Our working hypothesis was that, because NIr has almost direct access through the transparent cornea, lens, and humors the cells in the retina *in vivo*, it should be an effective protective and/or rescue agent, perhaps more so than for the SNc, a structure located deep in the brain and underneath the meningeal layers, cranium, skin, and hair. The retinae used for this study were from the same animals as those used in previous acute [4] and chronic [5] MPTP studies exploring the number of TH^+^ cells in the SNc.

## 2. Materials and Methods

### 2.1. Subjects

Male albino BALB/c mice (~20 g; ~8 weeks old; *n* = 80) were used. They were housed on a 12 hr light/dark cycle with unlimited access to food and water. All experiments were approved by the Animal Ethics Committee of the University of Sydney.

### 2.2. Experimental Design

**Figure 1:**
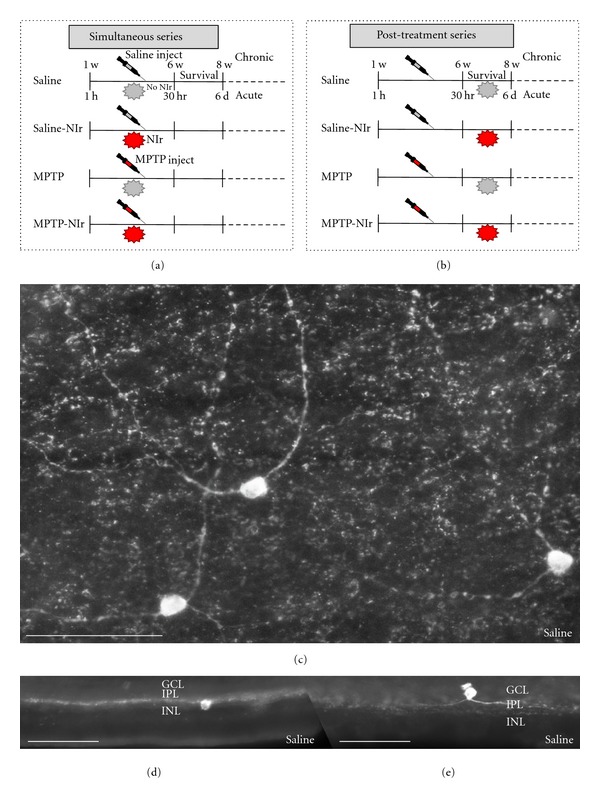
Outline of the different experimental groups used in this study, namely: Saline, Saline-NIr, MPTP, and MPTP-NIr, in either the simultaneous (a) or posttreatment (b) series of the acute and chronic models. Photomicrographs of TH^+^ amacrine cells in retinal wholemounts of the superior temporal region (c) and of the retinal edges ((d), (e)). The latter reveals the location of the TH^+^ somata and dendrites within the different layers. Most TH^+^ somata were located in the inner layers of the inner nuclear layer (c); very few were located in the ganglion cell layer (e). All images from Saline group of Ac-Sim case. Scale bars = 100 *μ*m.

An acute [4, 6, 7] and a chronic [5, 8] MPTP models were used in this study (Figures 1(a) and 1(b)). In each, NIr treatment was applied either at approximately the same time (simultaneous) or well after (posttreatment) the MPTP insult (Figures 1(a) and 1(b)). Hence, each model had two series, simultaneous (Acute-Simultaneous [Ac-Sim], Chronic-Simultaneous [Ch-Sim]) and posttreatment (Acute-Posttreatment [Ac-PT], Chronic-Posttreatment [Ch-PT]). Within each of these, there were four experimental groups, where mice received intraperitoneal injections of either MPTP or saline, combined with NIr treatments or not (Figures 1(a) and 1(b)). The different groups were: (1) Saline (*n* = 21): saline injections with no NIr (2) Saline-NIr (*n* = 19): saline injections with NIr (3) MPTP (*n* = 22): MPTP injections with no NIr (4) MPTP-NIr (*n* = 18): MPTP injections with NIr.

For the acute model, four (25 mg/kg injections; total of 100 mg/kg per mouse) MPTP or saline injections were made over a 30 hr period. After the last injection, mice were allowed to survive for six days. For the chronic model, mice had ten injections of MPTP (20 mg/kg per injection; total of 200 mg/kg per mouse) or saline combined with probenecid (250 mg/kg; decreases renal excretion of MPTP and hence maintains the effects of toxin during injection intervals), approximately three and a half days apart, over a five-week-period. After the last injection, mice were allowed to survive for three weeks. For both models, the dose regimes and survival periods were similar to those used by previous studies, including our own [4–11]. The survival periods were aimed to allow sufficient time for the MPTP to impart toxicity upon the dopaminergic cells. We did not observe any behavioural deficits in the mice after MPTP injection, although in some instances, the mice became quiescent immediately afterwards. However, these mice soon returned to normal activity, eating and grooming, within the next few hours.

For the NIr treatment, mice in the MPTP-NIr and Saline-NIr groups of each model (acute and chronic) were treated with 670 nm light from a light-emitting device (Quantum Devices WARP 10) as described previously [4, 5]. Briefly, for the simultaneous series of both models, mice had NIr treatment (the LED was held just above the mouse's head and in full view of their eyes and one cycle of 90 secs, estimated at 0.5 Joule/cm^2^, was applied) [4] ~15 mins after each MPTP or saline injection. Hence, for each MPTP insult there would be almost immediate potential therapeutic application. For the posttreatment series of both models, mice had NIr treatment approximately four days after the last injection. For the acute model, these treatments were spread over last two days of the survival period (total of four cycles), while for the chronic model, they were spread over three weeks (total of ten cycles). Hence, for this series, potential therapeutic application occurred well after the MPTP insult. For both models, these NIr treatment regimes were similar to that used by previous studies, in particular, those reporting changes after transcranial irradiation [4, 5, 12–17]. No behavioural or structural (e.g., in globe) deficits were evident after NIr treatment. The LED generated very little heat, and it did not cause any visible discomfort to the mice.

### 2.3. Immunohistochemistry

Following the survival periods, mice were anaesthetised with an intraperitoneal injection of sodium pentobarbital (60 mg/mL). They were then perfused transcardially with 0.1 M phosphate-buffered saline (PBS; pH 7.4), followed by 4% buffered formaldehyde. The retinae were removed and postfixed for ~20 mins in the same solution. Next, retinae were dissected free from other structures in the globe as wholemounts [18]. A deep cut was made in superior retina for orientation. Retinae were immersed in a solution of 1% Triton (Sigma) and 10% normal goat serum in PBS at room temperature for 1 hr. They were then incubated in antityrosine hydroxylase (TH; Sigma, 1 : 500) for ~48 hrs (at 4°C), followed by biotinylated anti-rabbit IgG (Bioscientific; 1 : 200) for ~4 hrs (at room temperature), and then Extravidin-FITC complex (Sigma; 1 : 200) for ~2 hrs (at room temperature). Between each incubation, retinae were washed in three changes of PBS. Retinae were mounted onto glass slides, coverslipped using Fluoromount (Sigma), and viewed under a fluorescence microscope. We found the FITC method far more sensitive than the peroxidase (and diaminobenzidine; DAB) method; FITC revealed the TH^+^ somata, together with their intricate dendritic plexus, more consistently across the entire retinal wholemount, from central to peripheral regions. The FITC method was certainly stable and durable enough for us to undertake a complete cell count and area analysis of each retina. For controls, sections were processed as described above, except that there was no primary antibody used. These control sections were immunonegative.

### 2.4. Analysis

Each retina was scanned systematically under the fluorescence microscope and the number of TH^+^ cells and retinal area were calculated with the aid of a stereological programme (StereoInvestigator, MBF Science). Every TH^+^ cell was plotted and total number was recorded; for the areas, the boundaries of each retina were traced and the programme calculated the area (mm^2^). For comparisons between groups (using GraphPad Prism programme), a oneway ANOVA test (*F* test; *P* value) was performed with a Tukey-Kramer multiple comparison test (*q* test; *P* value). Schematic diagrams and digital images were constructed using Adobe Photoshop and Microsoft PowerPoint programmes.

## 3. Results

The results that follow will be presented in four main parts: (i) morphology, (ii) retinal areas, (iii) number, and (iv) topography.

### 3.1. Morphology

Confirming previous studies [10, 19, 20], TH immunoreactivity in the mouse retina was seen in amacrine cells with large oval- or triangular-shaped somata (Figure 1(c)) located mainly in the inner part of the inner nuclear layer (Figure 1(d)); only one or two cells per retina were ever seen in the ganglion cell layer (Figure 1(e)). Most somata had one to two labelled primary dendrites that formed a rich overlapping plexus (Figure 1(c)) found in the inner plexiform layer (Figures 1(d) and 1(e)) [21]. In general, the morphology of TH^+^ amacrine cells was similar in all groups. 

### 3.2. Retinal Areas

**Figure 2:**
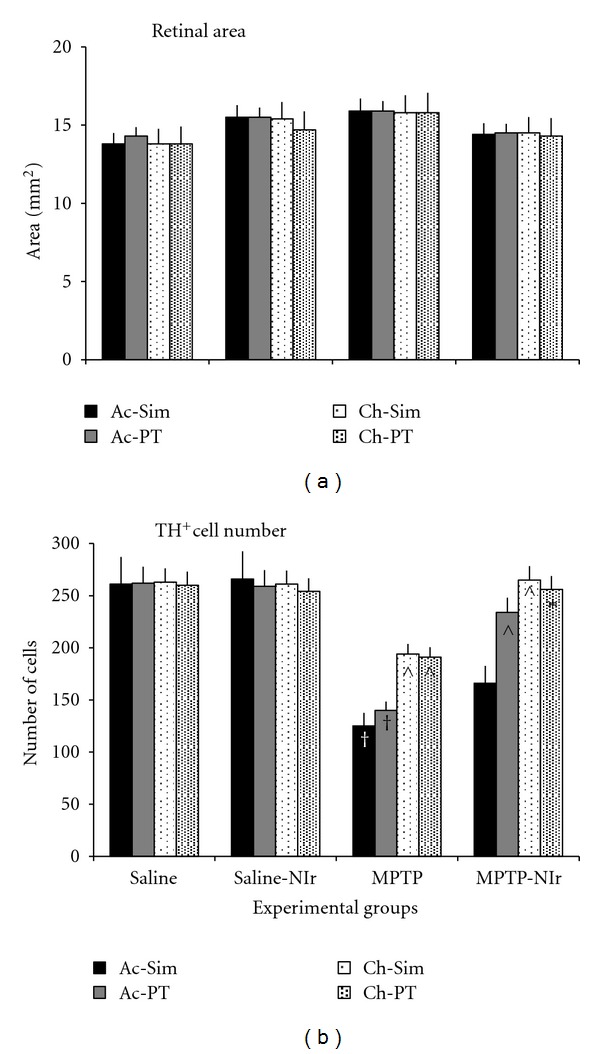
Graphs showing (a) the areas and (b) the number of TH^+^ amacrine cells in the retinae in the four experimental groups in the different cases (different shaded columns). Columns show the mean ± standard error of the total number of cells in each case. ^†^Represents *P* < 0.001, ^*∧*^represents *P* < 0.01 and *represents *P* < 0.05 significant difference in cell number; these symbols on the columns of the MPTP group represent differences from the Saline group of each case, while symbols on the MPTP-NIr group represent differences from the MPTP group of each case. There were clear increases in TH^+^ cell number in the MPTP-NIr group compared to the MPTP group in all cases, particularly the Ac-PT, Ch-Sim, and Ch-PT.

The graph in Figure 2(a) shows the retinal areas in the different experimental groups in each of the Acute-Simultaneous (Ac-Sim), Acute-Posttreatment (Ac-PT), Chronic-Simultaneous (Ch-Sim), and Chronic-Posttreatment (Ch-PT) cases. Overall, these values were similar to those reported previously for the retinal area of mice (~15 mm^2^) [19]. We found no significant difference between the retinal areas of the different cases (ANOVA test: *F* = 1.1; *P* = 0.4), indicating that our MPTP or NIr treatment had no impact on retinal area.

### 3.3. Number

The graph in Figure 2(b) shows the total number of TH^+^ cells in the retinae of the four experimental groups in each of the Ac-Sim, Ac-PT, Ch-Sim, and Ch-PT cases. Overall, the variations in number were significant (ANOVA test: *F* = 8.5; *P* < 0.0001). A more detailed analysis of TH^+^ cell number in the different cases will be considered in the paper.

For the Saline and Saline-NIr groups, TH^+^ cell number in the different cases were not significantly different (Tukey-Kramer test; *P* > 0.05). These values were comparable to those reported for normal mice by previous studies [10, 11, 19].

For the MPTP groups, TH^+^ cell number was reduced compared to the saline groups in all the cases. These reductions were significant (Tukey-Kramer test) in each of the Ac-Sim (*P* < 0.001Figure 2(b) ^†^), Ac-PT (*P* < 0.001; Figure 2(b) ^†^), Ch-Sim (*P* < 0.01; Figure 2(b) ^*∧*^), and Ch-PT (*P* < 0.01; Figure 2(b) ^*∧*^) cases. The reduction in TH^+^ cell number was greater in the acute (~50%) than in the chronic (~30%) cases (Figure 2(b)), and these differences were significant (*P* < 0.05). Conversely, no significant differences (*P* > 0.05) were found between the acute and between the chronic cases.

The number of TH^+^ cells in the substantia nigra pars compacta (SNc), from the same brains where the retinae were taken from, has been analysed too and full details of the results were published [4, 5]. Briefly, there was substantial TH^+^ cell loss in the SNc in both our acute (~60%) and chronic (~45%) MPTP models. In addition, there were also fewer TH^+^ terminals in the striatum, the major termination zone of the SNc axons, of the MPTP groups compared to the others. Hence, these features, together with the results on the retinae described above, confirm that our MPTP regime was effective.

In the MPTP-NIr groups, TH^+^ cell number was higher than in the MPTP groups in all the cases (~30%). These increases were significant (Tukey-Kramer test) in the Ac-PT (*P* < 0.01; Figure 2(b) ^*∧*^), Ch-Sim (*P* < 0.01; Figure 2(b) ^*∧*^), and Ch-PT (*P* < 0.05; Figure 2(b)*) cases, although not in the Ac-Sim case (*P* > 0.05; Figure 2(b)). When compared to the saline groups, TH^+^ cell number in the MPTP-NIr groups was reduced significantly only in the Ac-Sim case (Tukey-Kramer test; *P* > 0.01). In all the other cases, and unlike in the MPTP groups, TH^+^ cell number is not significantly different (*P* > 0.05) to the saline groups (Figure 2(b)).

In summary, TH^+^ cell number in the MPTP groups was reduced from the saline groups, particularly in the acute cases. In the MPTP-NIr groups, there were more TH^+^ cells compared to the MPTP groups in all cases, although to a lesser extent in the Ac-Sim case.

### 3.4. Topography

**Figure 3:**
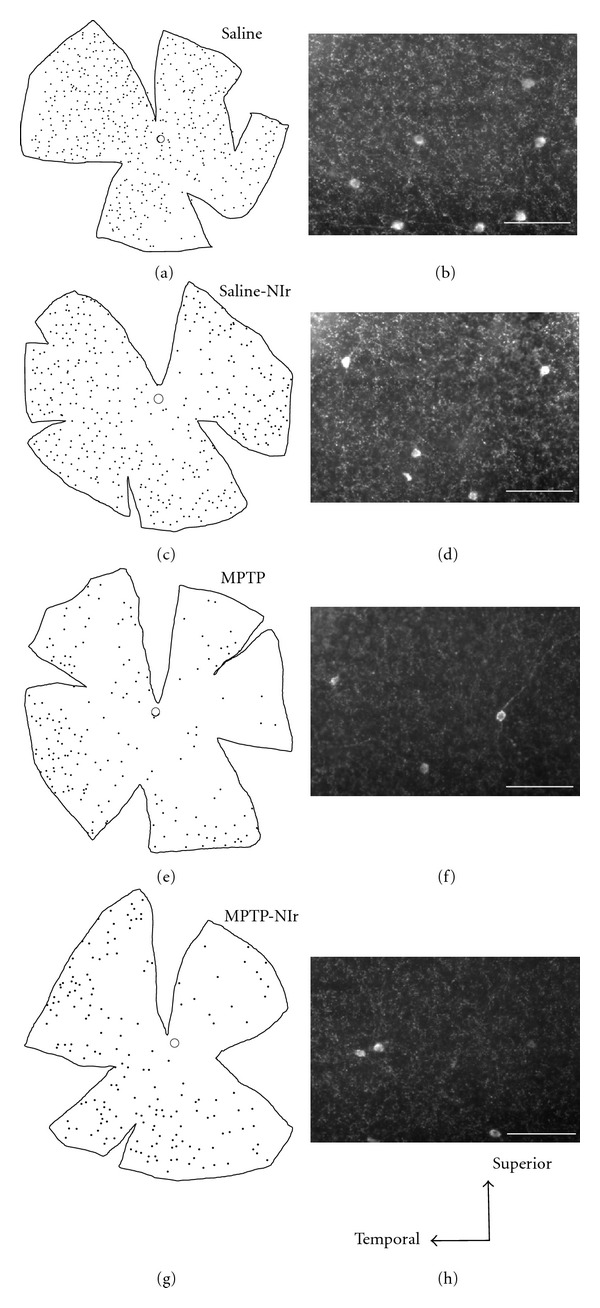
Schematic diagrams of maps ((a), (c), (e), (g)) and photomicrographs ((b), (d), (f), (h)) of TH^+^ amacrine cells in the retinae of Saline ((a), (b)), Saline-NIr ((c), (d)), MPTP ((e), (f)), and MPTP-NIr ((g), (h)) groups of the Ac-Sim case (this case shown because it had the most change after MPTP treatment). The photomicrographs are of a midregion of superior temporal retina in each case. In the saline control groups ((a)–(d)), TH^+^ cells were distributed relatively uniformly across the retina, but with a slight concentration in superior and temporal retina. In the MPTP and MPTP-NIr groups ((e)–(h)), there was no particular region of retina that was affected particularly after MPTP (or NIr) treatment. Scale bar = 100 *μ*m.

We examined the distribution of TH^+^ cells in the different cases as to determine whether the MPTP or NIr treatment affected one retinal region more than another.  Figure 3 shows maps and photomicrographs of TH^+^ cells in the representative retinae of Saline (Figures 3(a) and 3(b)), Saline-NIr (Figures 3(c) and 3(d)), MPTP (Figures 3(e) and 3(f), and MPTP-NIr (Figures 3(g) and 3(h) groups of the Ac-Sim case (this case shown because it had the most change after MPTP treatment). In all groups, TH^+^ cells were found in all retinal regions, but with more tendency to be located in superior and temporal retina [19, 21]. There was no particular region of retina that was affected greatly by either MPTP or NIr treatment, although there were fewer cells in the MPTP groups (see above in this case). In general, these patterns of distribution in each group were similar in the different cases.

## 4. Discussion

We had three main findings. First, there were fewer TH^+^ amacrine cells in the MPTP groups compared to the saline controls, particularly in the acute cases. Second, the magnitude of TH^+^ cell loss after MPTP insult was not as substantial as that seen in the SNc. Third, and importantly, there were more TH^+^ cells in the MPTP-NIr compared to the MPTP groups. Each of these issues will be discussed in this paper. First, a comparison with previous studies will be considered.

### 4.1. Comparison with Previous Studies

Previous studies have shown that NIr treatment offers *in vivo* protection to retinal photoreceptor cells against degeneration after exposure of either excessive illumination [22, 23] or methanol toxin [24]. We extend these findings by showing that NIr treatment protects another type of retinal cell, the TH^+^ amacrine cell, against MPTP insult. There have been many studies showing lower dopamine levels and TH immunoreactivity in the retinae of parkinsonian patients [25, 26] and MPTP-treated animals [10, 23, 27–30] compared to controls. Our present results support these findings. By contrast, Nagel and colleagues [11] using comparable dose regimes (150 mg/kg) and survival periods (7–14 days) have reported minimal loss of TH^+^ cells in the retinae of MPTP-treated mice. The reasons for these differences are not clear, although our results are similar in that TH^+^ cell loss was less in the retina than in the SNc.

### 4.2. MPTP Toxicity in the Retina and Comparison with SNc

Although some of our TH^+^ cell loss may be due to transient TH expression after MPTP insult [10], we suggest that the majority of the loss was due to cell death [6, 9, 31]. Many previous studies have shown that a loss of TH reflects overall cell survival. For example, MPTP insult has been shown to affect TH expression and then, after a prolonged period, generate cell death [31]. Furthermore, MPTP insult results in fewer Nissl-stained (and TH^+^) cells in the SNc indicating cell death [6, 9]. Nevertheless, whether transient expression or death (apoptotic or necrotic [32]), the important aspect of our study was that NIr treatment saved cellular TH expression during a period when MPTP treatment alone would have abolished.

The magnitude of TH^+^ amacrine cell loss after MPTP insult was greater in the acute cases (~50%) than in the chronic ones (~30%). Our chronic model delivered double the dose of MPTP (200 versus 100 mg/kg), but it was not as damaging to the amacrine cells as the acute insult delivered over a much shorter time period, 30 hours as against 5 weeks. Such findings have been reported previously by many studies. In the SNc, for example, acute insults generate up to 70% cell loss, while the chronic insults generate only about 50% [33, 34].

In the SNc, the reduction in TH^+^ cell number was ~15% greater than in the retina in both models [4, 5], indicating that the SNc cells are less resistant to MPTP insult than the retinal cells. Previous studies have reported similar findings, that the SNc cells (ventral sector) are more vulnerable to parkinsonian insult (i.e., MPTP insult, 6 hydroxydopamine lesion, idiopathic Parkinson disease) than other dopaminergic cell groups, for example, those in the ventral tegmental area, retrorubral field, dorsal sector of the SNc [1, 35, 36], zona incerta-hypothalamus, and periaqueductal grey matter [4, 5]. The factors that render retinal dopaminergic cells more resistant to MPTP insult than those in the SNc are not clear. For other dopaminergic cells it has been suggested that their levels of calbindin expression [35], dopamine transporter molecule (DAT) [37], and/or neuromelanin [38] may contribute to their greater resistance to toxic insult. The dopaminergic amacrine cells do not contain neuromelanin, but they have been reported to be calbindin^+^ [39] and DAT^+^ [37]. These two factors may contribute to their greater survival in the MPTP groups. We speculate also that melatonin, a hormone with antioxidant properties released by the nearby photoreceptors [40], has a role in rendering the amacrine cells more resistant to MPTP insult (see below). The issue of why some dopaminergic amacrine cells were more resistant to MPTP toxicity than others is not clear; the surviving cells appeared healthy and had no obvious change in their morphology (see Results). Perhaps these surviving cells contained more calbindin [39] and/or DAT [37] than those that had undergone cell death, and that these molecules served as neuroprotectants.

### 4.3. Patterns of Cell Protection and Rescue by NIr Treatment

There were more TH^+^ cells in the MPTP-NIr compared to the MPTP groups in all the cases, although to a lesser extent in the Ac-Sim case. There are three issues more to consider regarding this finding. First, despite the different types of intervening body tissue, whether transparent membranes of the globe or hair, skin, bone, and meninges, NIr treatment mitigated the MPTP insult just as effectively in the retina (~30%) and SNc (~35%) [4, 5]. Second, NIr treatment saved about the same number of retinal cells regardless of the nature of the parkinsonian insult, whether acute (~30%) or chronic (~25%). Third, NIr treatment saved about the same number of retinal cells whether applied at the same time (simultaneous) or well after (posttreatment) the MPTP insult (25–30%). NIr treatment was hence protective to healthy cells against insult, but also rescued damaged cells after the insult. Most of the protection was likely to have occurred in the simultaneous series, while the majority of the rescuing in the posttreatment series. A comparable pattern of protection and rescue has been noted in the SNc after NIr treatment [4, 5] and also after deep brain stimulation of the subthalamic nucleus in MPTP-treated monkeys [9].

The precise mechanism(s) that saved the TH^+^ amacrine cells from degeneration is not known. Many authors have suggested that NIr triggers intrinsic trophic factors that enhance cell survival, for example, by increasing ATP production and reducing reactive oxygen species in the mitochondria (see Introduction). In addition, we suggest that NIr treatment stimulated the local release of melatonin, a powerful antioxidant and cell saving agent [41, 42], from the retinal photoreceptors, that in turn, enhanced the survival of the dopaminergic amacrine cells [5, 43]. The local melatonin may have promoted mitochondrial activity and reduced oxidative stress in the amacrine cells, helping them survive the MPTP insult. Future studies may examine the effects of NIr treatment on retinal melatonin levels in normal and in parkinsonian cases.

## Abbreviations

Ac-Sim: Acute-simultaneous
Ac-PT: Acute-posttreatment
ATP: Adenosine-5′-triphosphate
Ch-Sim: Chronic-simultaneous
Ch-PT: Chronic-posttreatment
DAT: Dopamine transporter molecule
GCL: Ganglion cell layer
INL: Inner nuclear layer
IPL: Inner plexiform layer
LED: Light emitting device
MPTP: 1-Methyl-4-phenyl-1,2,3,6-tetrahydropyridine
NIr: Near-infrared light
PBS: Phosphate buffered saline
SNc: Substantia nigra pars compacta
TH: Tyrosine hydroxylase.
